# Private placements of equity and accessibility of bank loans

**DOI:** 10.1371/journal.pone.0281510

**Published:** 2023-03-15

**Authors:** Xin Song, Chao Liu, Zijie Ding, Chenchen Huang, Qiongyao Zhang

**Affiliations:** 1 University of Shanghai for Science and Technology, Shanghai, China; 2 Nanjing University of Finance & Economics, Nanjing, China; 3 Frostburg State University, Frostburg, MD, United States of America; 4 Robert Morris University, Moon Township, PA, United States of America; Al-Zaytoonah University for Science and Technology, STATE OF PALESTINE

## Abstract

This study investigates the changes in quantity and cost of bank loans after a private placement of common stocks by A-share listed companies in China from 2011 to 2021. This research is derived from the signaling theory and is based on a difference-in-difference design. Through propensity score matching, the sample comprises companies that placed equity privately in the experiment group and companies that did not place equity privately in the control group. We find evidence that the increase in bank loans slowed down, and the cost of bank loans increased after the private placement. The signaling effect of private placements is robust to various additional tests. Further analysis indicates that when state-owned enterprises place equity privately, their access to bank loans is not affected. When institutional investors participate in the private placement, the company’s access to bank credit does not go through significant changes. In addition, private placements by companies located in regions with higher levels of marketization of the financial market do not reduce the cost of bank loans.

## 1. Introduction

Equity private placements refer to listed companies selling a block of stocks privately to a limited number of investors. Private placements of common stocks are the prevailing venue of raising additional capital after the Initial Public Offering (IPO) by listed companies in China. For example, the total amount raised by private placements by A-share listed companies exceeded 1.5 trillion CNY in 2016, which accounted for more than 80% of funds raised by Seasoned Equity Offerings (SEOs) and was more than ten times what was raised by IPOs in the same year. Chinese companies use private equity placements to circumvent the regulatory restrictions on other forms of equity financing. For example, companies that plan to go IPOs or issue rights in SEOs must be a going concern with a track record of positive earnings. Private placements are less prohibitive and do not have those restrictions [1]. The monitoring effect theory posits that private places with big shareholders’ participation reduce the constraint of financing so that corporate innovations are encouraged [2]. The benefits of a private placement are primarily its ability to raise equity for companies. The downside of private placements is their tendency to be abused by large shareholders because of reduced regulatory requirements.

Listed companies in China often have a highly concentrated equity ownership structure. Protections for small shareholders are considered weak. Current literature on private placements focuses on the interest conflicts between big and small shareholders. For example, some studies investigate the schemes employed by big shareholders in funneling interests out of the company (e.g., [1,3]). We are interested in the impacts of private placements on creditors, including banks. In the Chinese financial market, banks often cannot access the necessary information to judge the credit risks of companies. Banks often depend on external signals [4]. Private placements provide a window into examining banks’ credit risk management mechanisms.

We select China’s A-share listed companies as the example in this study to investigate the relationship between private placements and bank loans. We find that private placements significantly affect the availability and costs of bank loans. Private placements offered at heavily(lightly) discounted prices see the more(less) significant restriction of bank loans. Private placements with institutional investors included are not followed by changes in bank loans granted to the company. We also discover that the signaling effect of a private placement is attenuated if the issuing company is a State-Owned Enterprise (SOE) or the company is located in a region with a developed financial market. When bank credit becomes tightened after a private placement, the company will likely use more commercial credit.

This paper makes a few potential contributions to the private placement literature. This paper documents the negative signaling effect of private placements on bank loans in China. Our analysis of the negative signal provides an additional opportunity to understand the interest competition between equity investors and creditors. Large shareholders will abuse their position to infringe on the interests of small shareholders without the monitoring of institutional investors and an effective financial market, which calls into question the effectiveness of the current investor protection mechanism put in place regarding approvals of private placements in China.

The remainder of the paper is organized as follows. Section 2 summarizes related literature and develops the research hypotheses. Section 3 discusses the research design. Results and analysis are reported in Section 4. Section 5 presents the results of robustness tests, and finally, Section 6 concludes the paper with a summary of our main findings.

## 2. Literature review and hypothesis development

### 2.1 Private placements and bank loans

[5] point out that enterprise investment decision-making should be independent of financing decision-making. Companies can acquire the fund they need at any time in a completely effective capital market. However, enterprises often face high financing constraints thanks to market frictions caused by information asymmetry [6]. Therefore, the financial market environment has a significant impact on the financing behavior of the company. China’s financial market is developing with room to grow, and banks often cannot effectively identify the potential default risks of listed companies. As a result, Chinese banks depend on external signals when making decisions on loans [4]. Private placements of common stocks affect the company’s equity structure, capital structure, stock price, and future performance. Private placements contain important, relevant information, serving as an external signal.

Chinese companies use private placements to raise capital because private placements face lower issuance hurdles than public offerings. In the Chinese market, private placements do not require the issuing company to be profitable, while positive earnings are required for public offerings. Private placements face fewer disclosure requirements and less oversight scrutiny from the regulatory authorities. A private placement is generally interpreted as a negative signal by the market [7]. In the meantime, the corporation tends to underperform in the stock market in both the short and long term after a high-valuation private placement [8]. [9] find that enterprises with worse financial fundamentals are more likely to place equity privately, and their performance is relatively worse after the private placement. [10] suggest that Japanese companies with poor operating results solve their financial crisis through private placements. According to [11] cost compensation hypothesis, companies that place equity privately have more severe asymmetric information problems. The discounted private placement price compensates for the information searching cost incurred because of the information asymmetry. Similarly [12], also show that companies with higher information asymmetry tend to place equity privately.

The literature suggests that the company’s long-term perspective is often dimmed after the private placement, sometimes referred to as the "SEO performance puzzle" [9,13]. The company faces a deteriorated credit financing environment after the private placement. When a borrower company’s performance worsens, banks will adjust their credit policy for new loans to impose higher interest rates or stop authorizing future loans [14]. The decline in performance after the private placement will inevitably add more uncertainty to the future of the enterprise.

Large shareholders’ interest-transferring behaviors send out another negative signal from the private placement. To purchase additional shares at a low price point, the major shareholders often manipulate the stock price to a lower level before the private placement. Studies have shown that the company often conducts negative earnings management before the private placement to drive down the stock price. But when institutional investors participate, the company conducts positive earnings management [15]. Ex-ante earnings management leads to subsequent deterioration of medium-term and long-term performance after the private placement [16].

Large shareholders’ interest-transferring behaviors around the private placement aggravate the negativity in the signal sent out by the private placement. In the context of China [17], find that big shareholders control the management to perform earnings management, which damages the value and reputation of the company. The market timing theory predicts that large shareholders can use their advantages on the board to profit from private placements. When the decision date of the board is used as the base date, large shareholders can buy low and sell high. In the early days of the private placement mechanism [18], warned of the risk of pricing failures. Later [19,20], show that the discount rate can be effectively restrained if the issuance date is strictly defined as the pricing base date. Capital infusion by large shareholders in the context of interest transfers generally generates little momentum for the corporation. The pricing standard of the first issuance date is associated with good operating results [21]. In addition, large shareholders also tend to transfer the wealth of listed companies by investing the company’s resources in so-called "inferior assets" that bring in poor returns or losses [22,23]. These bad investments reduce the value of the company’s assets, hurting the company’s chances of passing the bank’s credit screening.

Traditional corporate finance theory suggests that private placements can increase the proportion of equity capital in the company, which is positive for additional debt financing. However, as mentioned above, a private placement is often accompanied by significant information asymmetry, performance declines, earnings management, and injection of inferior assets in China’s capital market, which send negative signals to the capital market. Because banks in China rely on external signals, banks are likely to reduce their loans to the company after the private placement. Therefore, we hypothesize that:

**Hypothesis 1:** A private placement of equity will reduce the availability of bank loans to the company.

### 2.2 The private placement, discount rate, and bank loans

Private placements have two most salient features compared to seasoned equity offerings. It’s a private placement, with new common stocks issued to targeted investors, such as current major shareholders and institutional investors. Stocks are often sold at heavily discounted prices in a private placement. Thus, private placements could be used by large shareholders to transfer wealth and dilute smaller investors.

[24] report that the average price discount of China’s private placements is about 35%, which is greater than the discount rate between 8.7% and 20.4% in the U.S [25]. One argument for the discount is that the reduced price compensates for the higher information collection and processing costs by the investors [11]. The higher the cost of information collection and procession is, the greater the private placement discount is. An alternative explanation is that the discount compensates for the supervising cost that the investors incur. Investors often become large shareholders after participating in a private placement, which puts the investors in a position to monitor the company’s operation [45] more actively. Yet another hypothesis argues that the discount compensates for the lost liquidity because of stock resale restrictions after the private placement [26].

However, the aforementioned theories are not necessarily suitable in the Chinese context. Studies have shown that when a large shareholder participates in a private placement in China, the discount rate is significantly higher than when no large shareholders participate [27]. The large shareholders are the insiders with the information advantage, which does not fit the information cost theory. In China, the large shareholders are the actual controllers of the company before the private placement. The monitoring cost hypothesis is not applicable. Liquidity compensation is not sufficient to explain the discount, as studies show that large and shareholders easily sidestep the mechanism of stock transfer restrictions. For example, large shareholders can sell their existing shares in the secondary market before the private placement and then purchase additional shares from the private placement at discounted prices. This risk-free arbitrage renders the transfer restrictions toothless [28].

Price setting for private placements in China often involves opportunism and the transfer of interest by major shareholders [29,30]. The discounted price reflects the major shareholder’s encroachment on the small shareholders. Heavily discounted prices signal more significant interest conflicts between large and small shareholders. Banks will pick up the negative signal and tighten their credit to the company. Thus, we propose:

**Hypothesis 2:** Greater discount rates for private placement will lead to declines in bank loans and increases in loan costs.

### 2.3 Private placement and institutional investor

Institutional investors are important participants in China’s private placement market. Institutional investors have advantages over retail investors because they possess specialized knowledge and strong abilities to collect and process information. Institutional investors can alleviate the information asymmetry imposed by listed companies [31]. [32] found that the participation of institutional investors in China’s capital market can improve the information transparency of enterprises and alleviate the information asymmetry between the companies and investors. [33] also show that ownership by institutional investors can send a positive signal to the market about the company’s reasonable valuation and thus supports the price of stocks. Additional studies indicate that participation by institutional investors in private placements and other corporate governance initiatives brings about positive social effects [34].

[35] study institutional investors’ preference in participating in private placements. They find that a lockup period is associated with institutional investors’ long-term investment behavior, which services as valuation guidance for the company. Participation of institutional investors can introduce a major shareholder with the motivation and ability to monitor the management [45]. In addition, institutional investors can help the management make sound investment decisions, improve the efficiency of using capital, and suppress the decline in long-term performance due to ineffective use of funds raised from the SEO [16]. Similarly, participation by institutional investors has been found to improve the corporation’s performance in sustainability [36]. Banks could pick up the positive signal of participation by institutional investors, therefore, countering the negative signal of the private placement. This leads to our third hypothesis.

**Hypothesis 3:** Participation of institutional investors can mitigate the negative impact of private placements on securing bank loans.

## 3.Research design

### 3.1 Sample selection and data sources

We employ a Difference-in-Difference (DID) model to test the research hypothesis. The experiment group consists of private placements of common stocks of A-share listed companies in Shanghai and Shenzhen, China, from 2011 to 2021. Only the first private placement is included for multiple placements by the same company. The following firms are excluded from the sample: 1) S.T. and S.T.* companies; 2) financial and insurance companies; 3) A-Share listed companies that privately place B-shares or H-shares; 4) companies with a liability-asset ratio greater than 1; 5) companies whose financial data are missing; and 6) private placements accompanied with a public offering of equity. We winsorized all continuous variables at the 1% level. Companies in the control group are also A-share companies selected following the same procedures. We use the Propensity Score Matching (PSM) method to identify companies in the control group. The private placement data come from the Wind database and other financial data from the CSMAR database.

### 3.2 Model design and variable description

The following model (Model 1) is employed to test H1.


$$\mathrm{Loan}=\beta_{0}+\beta_{1}\mathrm{Treat}+\beta_{2}\mathrm{Post}+\beta_{3}\mathrm{Treat}\times \mathrm{Post}+\mathrm{Controls}+\varepsilon$$ (1)

In Model 1, the dependent variable Loan indicates the accessibility of bank loans to the company. *Loan* is measured by both the quantity (*T*.*D*.) and the price of bank loans (*Cost*). *T*.*D*. represents the change in total bank loans (short-term and long-term) between the beginning and the end of the year, scaled by revenues. *Cost* represents the interest expense of bank loans, proxied by the ratio of financing cost divided by revenues [37].

The dummy variable *Treat* equals 1 for a company that privately placed common stocks and equals 0 for a company without private equity placements. The dummy variable *Post* equals 1 for the year of the private placement and the subsequent years of a company in the experiment group, and equals 0 for the year before the private placement. We assign the same year of the private placement of the matched company in the experiment group to the control group so that *Post* has pseudo values for the control group. We are interested in β3, the coefficient of the interaction between *Treat* and *Post*.

We divide the full sample into two groups based on the discount rate and run Model 1 on the two groups in testing H2. We rank the discount rate of the private placement in descending order and categorize samples above the medium level as the high group and the rest as the low group. Based on whether institutional investors participate in the private placement, we separate the full sample into two subsamples, with and without institutional investors, to test H3. *Controls* represent the control variables in this study. Referring to the literature on corporate bank loans (e.g., [37,38], we include control variables such as Size, Age, Asset Structure (*PPE*), Leverage ratio (*Lev*), and Profitability (*ROA*) in the model. Table 1 summarizes the names and descriptions of the variables.

**Table 1:** Definitions and descriptions of major variables.

Variable Name	Variable Description
T.D.	The growth rate of bank loans. T.D. is calculated as (end term loan balance—beginning of the term loan balance)/ operating revenue
Cost	The Cost of bank loans. Cost is measured as finance cost/ operating revenue
Post	A dummy variable equals 1 for companies after their private placements of equity, otherwise 0
Treat	A dummy variable equals 1 if the private placement is implemented, otherwise 0
Discount	The discount rate of the private placement. Discount is calculated as (closing price before issue—issue price)/closing price before issue
Size	The size of the company. Size is measured by the natural log of the total assets of the company at the end of the fiscal year
Age	The number of years the company has been listed
Lev	The financial leverage of the company. Lev is calculated as debt /total assets
PPE	The structure of the company’s assets. PPE is calculated as (fixed assets + inventory)/ total assets
ROA	Return on Assets. ROA is calculated as net income/total assets
SOE	State Owned Enterprise. SOE is a dummy variable that equals 1 for state owned enterprises and 0 otherwise.
IND/Year	Industry fixed effect; year fixed effect

## 4. Results and analysis

Table 2 Panel A reports descriptive statistics of the variables. The mean value of *Cost* is 0.022, and the mean of *T*.*D*. is 0.011. The average discount rate of private placements is 0.623. Table 2 Panel B summarizes the results of the univariate analysis. The mean value of *T*.*D*. decreases from 0.015 to 0.006 after the private placement. The result is statistically significant at the 1 percent level. The mean value of *Cost* increases from 0.021 to 0.024 after the private placement, and the increase is statistically significant at the 1 percent level. Changes in the medium values of *T*.*D*. and *Cost* after the private placement show the same trend. After a private placement, a company’s ability to obtain bank loans declines, and the Cost of its bank loans increases, both suggesting the tightening of bank loans.

**Table 2:** Descriptive statistics and univariate analysis.

Panel A: Descriptive Statistics					
variable	sample	average value	standard deviation	minimum value	maximum value
Cost	14702	0.022	0.046	-0.061	0.328
TD	14702	0.011	0.16	-0.744	0.725
Discount	14702	0.623	0.485	0	1
Size	14702	22.533	1.347	18.932	26.382
Age	14702	14.193	6.577	2	31
Lev	14702	0.461	0.204	0.05	0.979
PPE	14702	0.381	0.185	0.004	0.8
ROA	14702	0.032	0.066	-0.323	0.259

Panel B: Univariate Analysis						
	mean test			median test		
	before the private placement	after the private placement	differences	before the private placement	after the private placement	differences
TD	0.015	0.006	0.009***	0.000	0.000	7.586***
Cost	0.021	0.024	-0.004***	0.01	0.012	14.947***

Note: Stars denote standard statistical significance (***p<0.01, **p<0.05, and *p<0.1, respectively).

### 4.1 Multivariate regression analysis

Based on the existing literature (e.g., [39]), we select company size (*Size*), company age (*Age*), the largest shareholder’s share (*Top*), State-Owned Enterprise (SOE), tangible assets (*PPE*), cash flow (*FCF*) and growth (*Grow*) co-variables to carry out the nearest neighbor matching. Table 3 reports the balance test results from the PSM matching process. All the differences of the co-variance are eliminated after the matching. The standard deviations are less than 10%, far below the recommended 20% threshold [40]. Panel D of Table 3 shows that the model cannot effectively predict whether a company places equity privately. The experiment and control groups do not have significant systematic differences after the match.

**Table 3:** Propensity score matching results.

Panel A							
Variable		Mean		% Reduction		T-Test	
		Processing Group	Control Group	% Bias	Absolute Value of Bias	T-Value	P-Value
Size	Unmatched	22.928	22.237	53.5		31.82	0.000
	Matched	22.923	22.934	-0.8	98.5	-0.43	0.666
PPE	Unmatched	0.3674	0.392	-13.1		-7.86	0.000
	Matched	0.368	0.371	-1.7	87.4	-0.92	0.359
Risk	Unmatched	0.027	0.027	-2.9		-1.74	0.082
	Matched	0.027	0.027	-0.7	77.1	-0.36	0.717
Cash	Unmatched	0.124	0.150	-19		-11.28	0.000
	Matched	0.124	0.127	-1.8	90.8	-1.05	0.293
ROA	Unmatched	0.025	0.037	-18.4		-11.10	0.000
	Matched	0.025	0.026	-1.5	91.7	-0.83	0.404
ROE	Unmatched	0.036	0.059	-14.8		-8.97	0.000
	Matched	0.038	0.041	-2	86.4	-1.02	0.309
Age	Unmatched	16.065	12.791	51.7		30.80	0.000
	Matched	16.044	16.062	-0.3	99.4	-0.17	0.866
Top	Unmatched	32.864	35.136	-15.1		-9.00	0.000
	Matched	32.9	32.701	1.3	91.2	0.74	0.459

Panel B					
	Pseudo R2	LR chi2	p>chi2	MeanBias	MedBias
Before the Private Placement	0.113	2271.9	0.000	23.6	16.8
After the Private Placement	0.000	4.86	0.772	1.3	1.4

We tested Model 1 with the matched data [41]. The results are presented in Table 4.

**Table 4:** Results of multivariate analysis.

	(1)	(2)	(3)	(4)
	TD	TD	Cost	Cost
Treat×Post	-0.017*** (-4.33)	-0.014*** (-3.61)	-0.002 (-1.54)	0.002** (2.36)
Treat	0.024*** (5.91)	0.013*** (3.12)	0.008*** (7.17)	-0.001 (-0.81)
Size		0.007*** (5.44)		0.000 (0.13)
Age		-0.001*** (-4.51)		0.000*** (2.83)
Lev		0.077*** (8.79)		0.075*** (33.96)
PPE		-0.037*** (-4.35)		0.013*** (6.20)
ROA		0.002 (0.08)		-0.097*** (-17.25)
SOE		-0.006* (-1.79)		-0.008*** (-10.46)
Cons	0.010 (0.16)	-0.123* (-1.86)	-0.004 (-0.24)	-0.006 (-0.36)
IND/Year	No	Yes	No	Yes
Adj R2	0.015	0.029	0.121	0.254
N	14,191	14,191	14,191	14,191

Note: *, **, and *** indicate significant levels of P < .1, .05, and.001 respectively.

*T*.*D*., the growth rate of new bank loans, is the dependent variable in columns 1 and 2. Column 1 does not have the control variables. The coefficient of the interaction term *Treat×Post* is 0.017 and is significantly negative at the 1 percent level. The control variables are added in Column 2, and the coefficient remains significantly negative at the 1 percent level. The growth rate of new bank loans decreases significantly after the private placement. Columns 3 and 4 report the regression results with the Cost of bank loans, *Cost*, as the dependent variable. Column 4 presents the complete model with the control variables. The coefficient of the interaction term is significantly positive at the 5 percent level. The Cost of bank loans significantly increases after the private placement. H1 is supported by the data. The effects of the control variables are consistent with the literature. Bigger companies have greater growth and smaller costs of bank loans. SOEs gain more loans from banks at lower costs compared with none SOEs.

Companies that privately placed equity are found to have issues with subsequent earnings declines, excessive earnings management, and funneling interests to large shareholders [3,9]. Those issues send negative signals to banks and other creditors. In less developed financial markets, those signals become an important factor in banks’ decision-making on extending credit and risk assessment.

We examine how the discount rate mediates between private placement and the changes in access to bank loans. We divide the experiment group into two groups by the medium of the discount rate of the private placement: the high-discount-rate group and the low-discount-rate group. The results are summarized in Table 5.

**Table 5:** Analysis results of subgroups.

	(1) high discount rate	(2) low discount rate	(3) high discount rate	(4) low discount rate
	TD	TD	Cost	Cost
Treat×Post	-0.014** (-2.51)	-0.015*** (-2.58)	0.003** (2.21)	-0.001 (-0.49)
Treat	0.009* (1.78)		-0.000 (-0.15)	
Size	0.008*** (4.68)	0.005** (1.97)	0.001 (1.42)	-0.000 (-0.64)
Age	-0.001*** (-3.86)	-0.001* (-1.84)	0.000 (0.50)	0.000*** (4.31)
Lev	0.066*** (5.89)	0.104*** (6.97)	0.074*** (25.07)	0.068*** (21.41)
PPE	-0.034*** (-3.09)	-0.044*** (-3.09)	0.019*** (6.40)	0.007** (2.25)
ROA	-0.007 (-0.24)	0.004 (0.12)	-0.091*** (-12.29)	-0.094*** (-11.51)
SOE	-0.003 (-0.80)	-0.010** (-2.02)	-0.007*** (-6.25)	-0.009*** (-7.90)
Cons	-0.136* (-1.90)	-0.094* (-1.83)	-0.023 (-1.22)	-0.007 (-0.63)
IND/Year	Yes	Yes	Yes	Yes
Adj R2	0.028	0.038	0.271	0.308
N	8,802	5,389	8,802	5,389

Note: *, **, and *** indicate significant levels of P < .1, .05, and.001 respectively.

For both *T*.*D*. and *Cost*, the high-discount-rate group has the statistically significant coefficient for the interaction term *Treat×Post*. For the indicator of the growth rate of bank loans, *T*.*D*., the coefficient is -0.014. For the indicator of the Cost of bank loans, *Cost*, the coefficient is 0.003. Private placements with discounted prices are the norm, but the market environment in China is different from that in developed countries. Chinese public companies tend to have issues of equity ownership concentration and be dominated by big shareholders. Big shareholders use private placements to transfer interests to specific parties. The greater the discount, the more interest is transferred. Greater discount rates are followed by more severe declines of company performance afterward [42,43]. Large discount rates exacerbate the negative signaling of private placements. Companies have more difficulties in accessing bank loans after private placements with large discount rates.

We investigate the impact of institutional investors on the relationship between private placements and the accessibility of bank loans. We segment the full sample into two groups, with institutional investors, and without institutional investors. The regression results are summarized in Table 6.

**Table 6:** Impacts of institutional investors.

	(1) participate	(2) not participate	(3) participate	(4) not participate
	TD	TD	Cost	Cost
Treat×Post	-0.014*** (-2.99)	-0.014* (-1.78)	0.003*** (2.72)	0.002 (1.06)
Treat		0.009 (1.26)		-0.000 (-0.09)
Size	0.006*** (3.29)	0.009*** (3.77)	-0.001** (-2.37)	0.002*** (2.97)
Age	-0.001** (-2.01)	-0.002*** (-4.07)	0.000** (2.53)	-0.000 (-1.06)
Lev	0.083*** (7.18)	0.077*** (5.34)	0.075*** (27.36)	0.072*** (19.44)
PPE	-0.051*** (-4.63)	-0.028** (-1.97)	0.020*** (7.55)	0.004 (1.20)
ROA	0.012 (0.41)	-0.017 (-0.45)	-0.094*** (-13.89)	-0.089*** (-9.48)
SOE	-0.009** (-2.24)	-0.001 (-0.11)	-0.008*** (-8.98)	-0.007*** (-5.13)
Cons	-0.122*** (-3.02)	-0.170** (-2.16)	0.001 (0.10)	-0.037* (-1.81)
Adj R2	0.032	0.033	0.279	0.292
N	8,474	5,717	8,474	5,717

Note: *, **, and *** indicate significant levels of P < .1, .05, and.001 respectively.

Columns 1 and 3 in Table 6 show that with institutional investors participating in the private placement, the coefficient of the interaction term is significant at the 1 percent level. With the involvement of institutional investors, the company’s access to bank loans changed after the private placement. Columns 2 and 4 reported the results when no institutional investors purchased shares in a private placement. The coefficients of *Treat×Post* are -0.014 and 0.002, respectively, and columns 2 is significant at the 10 percent level. It is evident that participation by institutional investors can not remediate the negative impact of private placements on accessing bank loans. Although the literature suggests that Institutional investors can mitigate information asymmetry using their own specialized advantages and information collection capabilities [44]. At the same time, the involvement of institutional investors introduces new major shareholders who have the ability and motivation to monitor the management [45]. The involvement of institutional investors improves corporate governance, serving as a good signal to the financial market. However, the findings of this paper suggest that the participation of institutional investors can not mitigate the negative impact of private placements on securing bank loans.

## 5. Robustness tests

### 5.1 Different time windows

Private placement is not a continuous financing behavior of firms. In order to avoid the confounding of other factors by the long window period before and after the differential model, this paper further limits the sample interval to four years before and after the private placement, and then re-tests the above three basic hypotheses to observe the impact of private placement on the credit screening mechanism of banks and the credit financing ability of firms. The results show that the aforementioned findings still hold.The regression results are shown in Table 7.

**Table 7:** Results of robustness tests.

	(1)	(2)	(3)	(4)	(5)	(6)
	full sample	full sample	high discount rate	low discount rate	institutional investors participate	institutional investors did not participate
VARIABLES	TD	Cost	Cost	Cost	Cost	Cost
Treat×Post	-0.015*** (-3.54)	0.002* (1.94)	0.003* (1.86)	-0.000 (-0.32)	0.003*** (2.84)	0.002 (0.97)
Treat	0.013*** (3.05)	-0.000 (-0.29)	0.001 (0.59)			0.000 (0.09)
Size	0.006*** (4.07)	0.000 (0.68)	0.001 (1.51)	0.000 (0.21)	-0.001** (-1.98)	0.002*** (3.51)
Age	-0.001*** (-3.89)	0.000* (1.85)	-0.000 (-0.03)	0.000*** (3.39)	0.000* (1.80)	-0.000 (-1.57)
Lev	0.089*** (8.77)	0.075*** (29.69)	0.074*** (22.69)	0.067*** (17.05)	0.074*** (22.42)	0.073*** (18.47)
PPE	-0.037*** (-3.79)	0.021*** (8.53)	0.028*** (8.66)	0.014*** (3.60)	0.028*** (9.08)	0.013*** (3.33)
ROA	0.024 (0.93)	-0.106*** (-16.59)	-0.100*** (-12.25)	-0.102*** (-10.41)	-0.099*** (-12.53)	-0.105*** (-10.30)
SOE	-0.009** (-2.45)	-0.008*** (-8.43)	-0.006*** (-4.78)	-0.009*** (-6.68)	-0.008*** (-7.11)	-0.007*** (-4.59)
Cons	-0.103 (-1.50)	-0.015 (-0.90)	-0.031 (-1.60)	-0.022* (-1.66)	-0.001 (-0.12)	-0.051** (-2.50)
IND/Year	Yes		Yes	Yes	Yes	Yes
Adj R2	0.030	0.265	0.288	0.307	0.279	0.316
N	11,182	11,182	7,298	3,884	6,204	4,978

Note: *, **, and *** indicate significant levels of P < .1, .05, and.001 respectively.

Column 1 and 2 report the result of the regression of the full sample.The interaction term Treat×Post coefficient is significant at the 1 percent level. Columns 4 and 6 report the low-discount-rate group’s regression results and the group without institutional investor participation, respectively.

### 5.2 Alternative explanation: Active or passive adjustment

An alternative explanation for the documented association between a private placement and consequently decreased bank loans and increased costs of bank loans is that the company needs less credit financing after the private placement. The company might actively reduce its bank loans to optimize its capital structure and reduce financial risks.

To investigate the alternative explanation, we examined the changes in the company’s external financing needs and financing constraints after the private placement. If external financing demands and financing constraints of the company increase after the private placement, the alternative explanation can be excluded. We use the difference between the actual growth rate and the endogenous growth rate to measure the external financing needs of enterprises. The actual growth rate is the asset growth rate; the endogenous growth rate is calculated as ROEt / (1-ROEt). Financing constraints are measured by the K.Z. and S.A. indices, which are widely used in the literature. The empirical results are presented in Table 8. Because the two regressions use different sets of independent variables, we only present the results of the main explanatory variables.

**Table 8:** Alternative explanations: Active adjustment or passive change.

	external financing demand	financing constraints	
	F.N.	SA index	K.Z. index
Treat×Post	-0.015** (-2.10)	0.024*** (5.65)	0.046 (1.53)
Treat	0.097*** (13.09)	0.006 (1.31)	-0.089*** (-2.88)
Cons	-0.262** (-2.17)	4.577*** (64.54)	10.148*** (20.48)
IND/Year	Yes	Yes	Yes
Adj R2	0.113	0.549	0.637
N	14,181	14,191	13,894

Note: *, **, and *** indicate significant levels of P < .1, .05, and.001 respectively.

After the private placement, the company’s need for external financing decreased substantially. Meanwhile, the financing restrictions faced by the company are more serious. The company’s financing needs are not fully met after the private placement. And thus, the possibility of the company intentionally reducing its credit financing can be ruled out.

### 5.3 SOEs and privately owned companies

In China, the financing environment faced by companies of different ownership rights varies significantly. State-Owned Enterprises (SOEs) have a natural advantage in access to bank loans [46]. Even in financial distress, soft budgetary constraints would enable an SOE to secure additional government funding [47,48]. Banks treat SOEs as preferable to privately-owned companies. Private companies typically have shorter and more expensive bank credit terms [49]. Table 9 displays the results of the regressions run separately on SOEs and private companies. SOEs do not experience tightened bank loans after the private placement. Private companies clearly face reduced availability and rising costs of bank loans after the private placement. This finding provides evidence that banks discriminate against private companies in their credit risk management.

**Table 9:** Impacts of ownership types.

	SOEs		Non-SOEs	
	TD	Cost	TD	Cost
Treat×Post	-0.009 (-1.63)	0.001 (0.40)	-0.020*** (-3.48)	0.005*** (3.23)
Treat	0.006 (0.96)	-0.001 (-0.78)	0.017*** (2.94)	-0.001 (-0.69)
Size	0.008*** (4.20)	-0.000 (-0.01)	0.008*** (3.70)	-0.000 (-0.28)
Age	-0.000 (-0.42)	0.000* (1.78)	-0.002*** (-4.65)	0.000 (1.08)
Lev	0.073*** (5.68)	0.070*** (21.92)	0.088*** (6.97)	0.081*** (25.72)
PPE	-0.032*** (-2.69)	0.021*** (6.92)	-0.051*** (-4.13)	0.006* (1.88)
ROA	-0.089** (-2.45)	-0.072*** (-8.05)	0.054* (1.90)	-0.101*** (-14.07)
Cons	-0.133* (-1.85)	-0.016 (-0.91)	-0.173*** (-3.46)	-0.017 (-1.36)
IND/Year	Yes	Yes	Yes	Yes
Adj.R2	0.030	0.284	0.042	0.271
N	7,156	7,156	7,035	7,035

Note: *, **, and *** indicate significant levels of P < .1, .05, and.001 respectively.

### 5.4 Different levels of marketization of the local financial market

The marketization level of the regional financial market is an important factor that affects the bank’s credit decision. When the marketization level of the financial market is low, banks face more severe information asymmetry and often rely on external signals to make loan decisions [4]. In a less developed market, the signaling effect of a private placement is stronger, and its impact on the bank’s credit management mechanism is more potent. Therefore, we further examine the impact of the marketization level of the financial market on corporate credit financing. We adopt the "marketization index of the financial industry" from [50]. Based on the company’s headquarter location, we divide the full sample into two groups: one group located in the highly marketized financial market and the other in the less marketized financial market. We compared these two groups and presented the results in Table 10.

**Table 10:** Differences in the levels of financial market development.

	Higher level of marketization		Lower level of marketization	
	TD	Cost	TD	Cost
Treat×Post	-0.015*** (-2.63)	0.000 (0.28)	-0.016*** (-2.72)	0.004*** (2.65)
Treat	0.011* (1.93)	0.002 (1.60)	0.014** (2.32)	-0.003* (-1.86)
Post	0.006*** (3.10)	-0.001** (-2.16)	0.009*** (4.60)	0.001** (2.32)
Size	-0.001*** (-3.12)	0.000 (0.70)	-0.001*** (-3.02)	0.000*** (2.64)
Age	0.088*** (7.04)	0.063*** (24.26)	0.067*** (5.29)	0.087*** (24.28)
Lev	-0.038*** (-3.13)	0.015*** (5.74)	-0.042*** (-3.39)	0.008** (2.44)
PPE	-0.024 (-0.79)	-0.075*** (-11.70)	0.031 (0.93)	-0.112*** (-12.03)
ROA	-0.008* (-1.71)	-0.003*** (-3.35)	-0.003 (-0.66)	-0.014*** (-10.72)
SOE	-0.154*** (-3.03)	0.003 (0.26)	-0.150** (-2.07)	-0.024 (-1.19)
Cons	-0.015*** (-2.63)	0.000 (0.28)	-0.016*** (-2.72)	0.004*** (2.65)
IND/Year	Yes	Yes	Yes	Yes
Adj.R2	0.037	0.266	0.034	0.279
N	7,128	7,128	7,063	7,063

Note: *, **, and *** indicate significant levels of P < .1, .05, and.001 respectively.

For companies in regions with a higher level of marketization of the financial market, their bank loans did not change significantly after the private place, regarding the cost of bank loans. In the second group, with a lower level of financial market marketization, companies had significantly lower quantity and higher costs of bank loans after the private placement. We interpret the difference as banks are more dependent on external signs to judge their credit risk in a less developed financial market. A private placement is picked up by the banks as a significant negative signal. Private placement can improve the negative impact on bank lending in a more developed financial market.

## 6 Summary and conclusions

Rooted in the signaling theory, we use the PSM-DID design to investigate the influence of private placement on banks’ credit risk management mechanisms in China. We found that private placements lead to a tightening of bank loans, leading to a significant reduction in companies’ edit financing from banks, as evidenced by a decline in the growth rate of new loans and a significant increase in loan costs. We investigated two additional signals: the participation of institutional investors in the private placement and the discount rate of the private placement price. The greater the price discount rate is, the more restrictive the bank loans become. This negative signaling effect of a price placement can remain intact when institutional investors are involved.

The findings in this study hold to several robustness tests. We changed the time window on the periods before and after the private placements, and the findings stand. We excluded the alternative explanation that the company intentionally reduced its bank loans after the private placement. Companies use commercial credit substitutes for bank loans when bank loans’ growth slows down after the private placement. Further analysis indicates that the signaling effect is only significant among private companies and not significant among SOEs. The signaling effect is more effective in locations with less developed financial markets.
